# Comparison of Instrumental Activities of Daily Living assessment by face-to-face or telephone interviews: a randomized, crossover study

**DOI:** 10.1186/s13195-020-00590-w

**Published:** 2020-03-13

**Authors:** Virginie Dauphinot, Nawèle Boublay, Claire Moutet, Sarah Achi, Anthony Bathsavanis, Pierre Krolak-Salmon

**Affiliations:** 1 Memory Clinical and Research Center of Lyon (CMRR), Lyon Institute For Elderly, Hospices Civils de Lyon, Lyon, France; 2 Hôpital des Charpennes, 27 rue Gabriel Péri, 69100 Villeurbanne, France; 3 Université Lyon 1, Lyon, France; 4 INSERM, U1028; CNRS, UMR5292; Lyon Neuroscience Research Center, Brain Dynamics and Cognition Team, F-69000 Lyon, France

**Keywords:** Activity of daily living, Neurocognitive disorders, Memory, Alzheimer’s disease

## Abstract

**Background:**

The functional autonomy assessment is essential to manage patients with a neurodegenerative disease, but its evaluation is not always possible during a consultation. To optimize ambulatory autonomy assessment, we compared the Lawton Instrumental Activities of Daily Living (IADL) questionnaire collected by telephone and face-to-face interviews.

**Methods:**

A randomized, crossover study was carried out among patients attending a memory clinic (MC). The IADL questionnaire was collected for patients during telephone and face-to-face interviews between nurses and patients’ caregivers. The agreement between the two methods was measured using the proportion of participants giving the same response, Cohen’s kappa, intraclass correlation (ICC) coefficient, and Bland and Altman method. The associations between patients’ characteristics, events occurring between the two assessments, and agreement were assessed.

**Results:**

Among the 292 patients (means ± SD age 81.5 ± 7, MMSE 19.6 ± 6, 39.7% with major neurocognitive disorders) analyzed, the proportion of agreement between the two modes was 89.4% for the total IADL score. Weighted kappa coefficient was 0.66 and ICC score was 0.91 for total IADL score. The mean difference between the IADL score by telephone or face-to-face was 0.32. Overall, 96.9% of measures lay within the 95% limits of agreement. The occurrence of fall was less likely associated with the probability to lie within the 95% limits of agreement (OR = 0.07 [0.02–0.27]).

**Conclusion:**

The administration of IADL by telephone with the caregiver appears to be an acceptable method of assessment for MC patients compared to face-to-face interview. The events such as falls which could occur in a time close to the evaluation should be reported.

**Study registration:**

ClinicalTrials.gov, NCT02654574. Retrospectively registered: 13 January 2016

## Background

The assessment of functional autonomy is an essential step in evaluating and caring patients in memory clinics (MCs), and it is included in the diagnosis procedure to determine the stage of diseases. The major neurocognitive disorder (NCD) is thus defined as a syndrome characterized by a decline in cognitive functions severe enough to interfere with patient’s ability to perform everyday activities, while in mild NCD, the patient’s abilities are not significantly impacted [1]. Although there is no standard measurement of functional status, and information on the metric proprieties of the Lawton Instrumental Activities of Daily Living (IADL) questionnaire is limited [2], it is commonly used in MC during face-to-face interview [3]; it assesses patients’ ability to perform daily tasks considered as complex activities for which different cognitive processes are involved. The Lawton IADL questionnaire has also been identified as a scale frequently used to measure functional outcome in Alzheimer’s disease [4]. However, in current practice, the systematic collection of the IADL questionnaire can be problematic due to limited time available for the medical staff or the patient’s caregivers. At the same time, minimization of missing data is essential for patient management but also to ensure a sufficient quality of data for research. The collection of data through other modes of administration, such as telephone interview, has been previously proposed to achieve these goals, and, while several studies have compared telephone and telehealth administration to face-to-face interviews for different cognitive questionnaires, none has studied the Lawton IADL questionnaire [5–8]. Such an evaluation is required as it is reported that the mode of administration could influence the quality data. For instance, in the study reported by Bowling et al., questionnaire administration by telephone was considered to be more cognitive burdensome for the respondents, to provide less information in the responses, and to be less preferred by the respondents compared to face-to-face interview [9]. We therefore conducted a randomized crossover study to measure the agreement between telephone and face-to-face administration of the IADL questionnaire. Furthermore, we assessed whether patient characteristics may impact the degree of agreement.

## Methods

### Study design

We carried out a randomized, open-label, crossover study, with two study periods (sequences AB/BA) and four assessment points. A wash-out period of 30 days was chosen to avoid remembrance of previous answers.

The study was conducted in the same context of the MEMORA cohort that aims to study the relationship between patient characteristics and functional autonomy change over time among patients attending a MC [10].

### Participants and setting

Eligibility criteria for participants were patients attending a memory consultation for the first time at the MC for a diagnostic work-up, aged 50 years or older, living at home, accompanied by an informal caregiver, and who agreed to participate in the study. Exclusion criteria were patients whose caregiver did not wish to participate in the study, patients whose caregiver did not provide a telephone contact, and patients for whom the health status would require institutionalization during the period of the study. The study was conducted at a MC of the Memory Research Center of Lyon (France), between November 2014 and April 2016.

### IADL questionnaire and modes of administration

The questionnaire used to assess the level of functional independence (or dependence) was the French version of the Lawton IADL including the 8 items: ability to use the telephone, to go shopping, to prepare food, to do housekeeping, to do personal laundry, to use transportation, to be responsible for taking medications, and to handle finances [2]. The questionnaire answers have been considered as a total score ranging from 0 (dependent) to 8 (independent), as well as 2 sub-scores of 4 based on previous research [11, 12]. The first sub-score includes the items concerning telephone, transportation, medications, and finances. The second sub-score includes the other items. In addition, each item of the IADL questionnaire has been scored as binary variables (1: ability to conduct the activity, 0: no ability).

The IADL questionnaire was collected for the same patients using two modes of administration: by telephone and face-to-face interviews. Both interviews consisted of a collection of answers given by the caregiver to the nurses trained for this procedure. The questionnaire was identical in both modes of administration, the questions were asked following the same order, and the nurses had to read the questionnaire exactly as it was written to ensure similar conditions for data collection.

Potentially eligible patients were selected from the list of scheduled appointments in the MC. A letter to inform both the patient and caregiver of the possibility to participate in the study was sent along with the appointment confirmation letter for the memory consultation. The nurse contacted the patients and caregivers depending on the telephone number available, presented the study, checked the eligibility criteria, and asked whether they agreed to participate. If they did so, they were assigned randomly to one of the two branches of the study. For the patients in the first branch, the telephone appointment was planned with the caregiver 1 month after the memory consultation. For the patients in the second branch for whom the telephone appointment was the first administration of the IADL questionnaire, a telephone appointment was planned with the caregiver 1 month before the memory consultation. In case the call did not succeed, the nurse was to try again up to 4 times.

### Patients and study characteristics

Additionally to the IADL questionnaire, we considered the following patients’ characteristics collected using the electronic case report form (eCRF) of the MEMORA study: age, sex, marital status, relationship between the caregiver and the patient education level, cognitive status, etiology, and the Mini-Mental State Examination (MMSE) score ranging from 0 to 30 and evaluating the overall cognitive performance. Details of the collection of the data in the MEMORA study are available elsewhere [10]. These data were collected at different times, i.e., age, sex, marital status, and the relationship between the caregiver and the patient were collected at inclusion of the study (at randomization), while the other data were collected during the face-to-face interview at the MC. Indeed, for organizational reasons, it was not possible to schedule an additional visit for patients whose telephone interview was scheduled first, since face-to-face interview would take place 1 month later.

Additional information was collected at the second interview using a paper CRF by the nurses, either by face-to-face interview or telephone in order to detect possible changes between the two measures: change of caregiver respondent between the 2 assessments and change of nurse who administered the questionnaire, and the following events: admission to the emergency department, hospitalization, occurrence of a fall, change of living place, and occurrence of death in the family. The number of calls needed to reach the participants, the duration of the telephone interview, and the reason why the questionnaire could not be administered after randomization were also collected.

### Randomization

After obtaining oral consent for participation, randomization was performed using a computer-generated list (Microsoft Excel 2010). It was a centralized and restricted randomization with an allocation ratio of 1:1 in a fixed block of 4 individuals. The nurses, who enrolled the patients, assigned each of them to one of the branches according to the random allocation and successively in the order of inclusion.

### Sample size

The sample size was calculated using the STATA software version 13 (StataCorp. College Station, TX) (SSQDL function). For Cohen’s kappa coefficient of 0.8, a proportion of patients dependent for at least 2 items of the IADL questionnaire at 50%, with a power of 80% and a risk alpha of 0.05, the sample size required was 138 patients per branch. With an expected loss to follow-up or missing value proportion of 30%, the total sample size was estimated to be 197 patients per branch.

### Statistical analysis

A flowchart has been made to describe the recruitment of the population. The characteristics of patients who had completed both the first and second assessments were compared to those of patients who had completed only the first assessment, using the Pearson *χ*^2^ test or Fisher’s exact test to compare proportions or independent Student’s *t* test to compare means. The characteristics of the final study population were compared between the branches. Characteristics of the patients were summarized using mean ± standard deviation (SD) or number of patients (percentage), as appropriate. The duration of the telephone interview was presented as mean ± SD in minutes.

In the main analysis, the extent of agreement between the two modes of administration was measured using the following statistics: the proportion of participants who gave the same response for both modes of administration (the proportion of patients according to the different cases was compared using the McNemar chi-squared test); the linearly weighted Cohen’s kappa coefficient [13] for the total IADL score and for the 2 sub-scores of IADL; the unweighted Cohen’s kappa coefficient [14] for each item of the IADL score (coded as binary variables) and each level of independence according to the total IADL score, e.g., autonomy for 8 abilities vs. 0 and autonomy for ≥ 7 abilities vs. less; the intraclass correlation coefficient (ICC) (“two-way mixed effects, absolute agreement, multiple raters/measurement” form) [15] for the total IADL score; and the Bland and Altman analysis [16]. These analyses were conducted in all the study population and separately in both branches.

The level of agreement according to kappa coefficients was interpreted as 0–0.2: none, 0.21–0.39: minimal, 0.4–0.59: weak, 0.6–0.79: moderate, 0.8–0.9: strong, and ≥ 0.9: almost perfect [17]. The level of agreement based on the ICC was interpreted as < 0.5: poor, 0.5–0.75: moderate, 0.75–0.9: good, and ≥ 0.9: excellent [18].

In an additional analysis, the associations between the patients included in the 95% limits agreement vs. those outside, and the patient characteristics were assessed using logistic regression models. The results were presented as odds ratios and 95% confidence intervals (OR [95% CI]).

Missing data was not replaced. *p* values less than 0.05 were considered statistically significant. Analyses were performed using SPSS (Statistical Package for the Social Sciences) version 19.0 for Windows (SPSS Inc., Chicago, IL, USA).

## Results

### Description of the study and characteristics of the study population

**Fig. 1:**
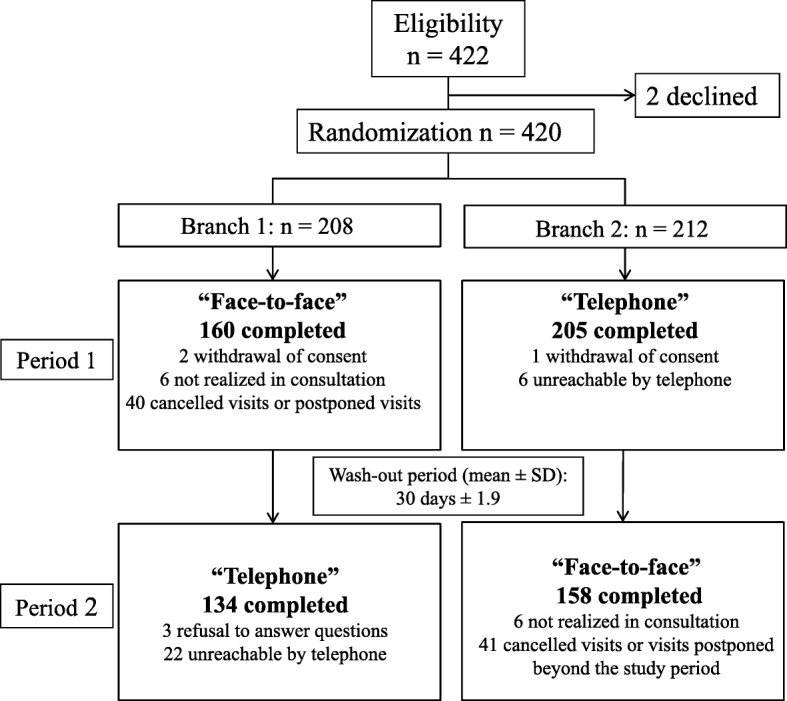
Study flowchart

Overall, 420 participants were selected and randomized (Fig. 1). Among them, the IADL could be collected at the first assessment for 365 patients. After the second assessment, the IADL was available for both modes of administration for 292 patients (69.5% of the selected participants). The interval between the two IADL assessments was 29.8 ± 1.9 days (29.7 ± 2.2 in branch 1 and 30.1 ± 1.5 in branch 2). The majority of incomplete data at the second assessment was explained by unreachable participants in branch 1 (face-to-face then telephone) and by canceled or postponed visits in branch 2 (telephone then face-to-face). The proportion of unreachable participants by telephone was higher in branch 1 (*n* = 22/160, 13.8%) than in branch 2 (*n* = 6/212, 2.8%).

The duration of the telephone interview including the IADL assessment was collected for 255 participants. In the total sample, the telephone interview lasted 7 ± 3.7 min (range 3–27 min); for 127 participants of the branch 1, the telephone interview lasted 7.4 ± 3.9 min (range 3–27 min), and for 128 participants of the branch 2, the telephone interview lasted 6.7 ± 3.5 min (range 3–21 min).

Patients with IADL measures with the 2 modes of administration had similar baseline profile than those without IADL measure at the second assessment in terms of age, sex, etiology, MMSE, and IADL; education level, marital status, and cognitive status were less frequently identified as the data could not be collected during the face-to-face interview (Supplementary files. Table 1). There was a higher proportion of drop-out in branch 2 (22.9%) than in branch 1 (16.3%).

The study population (mean ± SD age 81.5 ± 6.9 years) was characterized by a higher proportion of women (58.9%) compared to men (41.1%), a higher proportion of patients with less than 12 years of education (59.3%) while 18.2% had more than 12 years of education (education level was unknown for 22.6% of the sample), a higher proportion of patients married or in a couple (42.8%) while 29.1% were single (Table 1). A majority of patients had major NCD (39.7%), 20.9% had mild NCD, and 27.7% isolated memory complaint. A majority of patients had awaiting etiological diagnosis (62.3%), and 22.3% had probable Alzheimer’s disease (22.3%). Mean MMSE was 19.6 ± 6.1, mean IADL by telephone was 3.6 ± 2.2, and mean IADL by face-to-face interview was 3.3 ± 2.2. Involved person as a caregiver was mainly the child of the patient (60.3%) followed by the spouse (32.5%). Patients’ characteristics were not significantly different between the two branches. There was a higher proportion of patients with falls (9.7%) and change of living place (3%) between the 2 assessments in branch 1 compared to branch 2 (3.2%, 0% respectively). The mean number of calls to reach the participants was higher in branch 1 (1.5 ± 0.8) compared to branch 2 (1.1 ± 0.3).

**Table 1:** Characteristics of the patient sample and according to study branch

	Total	Branch 1 (***n*** = 134)	Branch 2 (***n*** = 158)
**Age (year)**, mean ± SD	81.51 ± 6.95	81.64 ± 6.95	81.41 ± 6.96
**Sex**, *n* (%)			
Female	172 (58.9%)	82 (61.19%)	90 (56.96%)
Male	120 (41.1%)	52 (38.81%)	68 (43.04%)
**Education**, *n* (%)			
≤ 12 years	173 (59.25%)	79 (58.96%)	94 (59.49%)
> 12 years	53 (18.15%)	22 (16.42%)	31 (19.62%)
Unknown	66 (22.6%)	33 (24.63%)	33 (20.89%)
**Marital status**, *n* (%)			
Married/in couple	125 (42.81%)	58 (43.28%)	67 (42.41%)
Single/widowed	85 (29.11%)	44 (32.84%)	41 (25.95%)
Divorced/separated	12 (4.11%)	6 (4.48%)	6 (3.8%)
Others/unknown	70 (23.97%)	26 (19.4%)	44 (27.85%)
**Cognitive status**, *n* (%)			
Isolated memory complaint	81 (27.74%)	41 (31.6%)	40 (25.32%)
Mild neurocognitive disorders	61 (20.89%)	23 (17.16%)	38 (24.05%)
Major neurocognitive disorders	116 (39.73%)	56 (41.79%)	60 (37.97%)
No neurocognitive disorders/unknown	34 (11.64%)	14 (10.45%)	20 (12.66%)
**Etiology**, *n* (%)			
Probable Alzheimer’s disease	65 (22.26%)	34 (25.37%)	31 (19.62%)
Other neurological diseases	45 (15.41%)	19 (14.18%)	26 (16.46%)
Awaiting diagnosis	182 (62.33%)	81 (60.45%)	101 (63.92%)
**MMSE** (*n* = 250), mean ± SD	19.58 ± 6.14	19.58 ± 6.11	19.57 ± 6.19
**IADL by telephone**, mean ± SD	3.57 ± 2.18	3.40 ± 2.21	3.72 ± 2.16
**IADL by face-to-face**, mean ± SD	3.25 ± 2.18	3.13 ± 2.05	3.35 ± 2.29
**Relationship between the caregiver and the patient at baseline**			
Spouse	95 (32.53%)	44 (32.84%)	51 (32.28%)
Child	176 (60.27%)	83 (61.94%)	93 (58.86%)
Other	21 (7.19%)	7 (5.22%)	14 (8.86%)
**Change of respondent between the 2 assessments**			
No	284 (97.26%)	127 (94.78%)	157 (99.37%)
Yes	8 (2.74%)	7 (5.23%)	1 (0.63%)
**Events between the 2 assessments**			
Admission to emergency department (ref. no entry)	11 (3.77%)	7 (5.22%)	4 (2.53%)
Occurrence of an hospitalization (ref. no hospitalization)	14 (4.79%)	9 (6.72%)	5 (3.16%)
Occurrence of a fall (ref. no fall)	18 (6.16%)	13 (9.70%)	5 (3.16%)
Occurrence of a death in family (ref. no occurrence)	2 (0.68%)	1 (0.75%)	1 (0.63%)
Change of living place (ref. no change)	4 (1.37%)	4 (2.99%)	0
**Change of nurse between the 2 assessments**			
No	280 (95.89%)	125 (93.28%)	155 (98.10%)
Yes	12 (4.11%)	9 (6.72%)	3 (1.90%)
**Number of calls**, mean ± SD	1.29 ± 0.61	1.52 ± 0.78	1.08 ± 0.30
1	230 (78.77%)	84 (62.69%)	146 (92.41%)
2	43 (14.73%)	32 (23.88%)	11 (6.96%)
3	16 (5.48%)	15 (11.19%)	1 (0.63%)
4	3 (1.03%)	3 (2.24%)	0

### Agreement between the 2 modes of administration of the IADL

The weighted kappa coefficient was 0.66 for the total IADL score, 0.69 for the first sub-score, and 0.62 for the second sub-score, reflecting moderate agreement between the 2 modes of administrations of IADL questionnaire (Table 2). The analysis by item of the IADL found kappa coefficients ranging from 0.47 (ability to handle finances) to 0.75 (mode of transportation), indicating a weak to moderate agreement depending of the items, and kappa coefficients ranging from 0.59 (≥ 1 IADL vs. less and ≥ 0 IADL vs. more) to 0.71 (≥ 4 IADL vs. less and ≥ 5 IADL vs. less) according to the level of autonomy indicating a weak to moderate agreement between the 2 modes. The ICC score for total IADL scores by telephone and face-to-face was 0.91 (95% CI of ICC score 0.89–0.93) for the total study population, 0.91 (0.88–0.94) in branch 1 and 0.91 (0.89–0.94) in branch 2. The proportion of agreement between the two modes was 89.4% for total IADL and ranged from 75% (ability to handle finances) to 91.8% (ability to use telephone) according to the item of the IADL and 83.9% (autonomy for 3 IADL items vs. less) to 96.6% (autonomy for 8 IADL items vs. 0) according to the level of autonomy. The results were of the same order of magnitude whether the IADL questionnaire was administered by telephone or face-to-face first (Supplementary files. Table 2).

**Table 2:** Description of agreement between the 2 modes of administration of the IADL questionnaire for the total patient sample

	Total (***n*** = 292)						
	***n*** −/− (***a***)	***n*** −/+ (***b***)	***n*** +/− (***c***)	***n*** +/+ (***d***)	Agreement* %	***K***^‡^	***p*** value^**†**^
IADL (/8)	–	–	–	–	89.38%	0.66	< 0.001
Sub-score 1 (/4), i.e., phone, transportation, medications, finance	–	–	–	–	89.47%	0.69	< 0.001
Sub-score 2 (/4), i.e., shopping, food, housekeeping, laundry	–	–	–	–	85.87%	0.62	< 0.001
**IADL by item (autonomy: yes vs no)**							
Ability to use telephone	33	10	14	235	91.78	0.69	0.54
Shopping	212	27	15	38	85.62	0.56	0.09
Food preparation	209	31	8	44	86.64	0.61	< 0.001
Housekeeping	66	41	23	162	78.08	0.52	0.03
Laundry	137	30	16	109	84.25	0.68	0.054
Mode of transportation	170	23	11	88	88.36	0.75	0.06
Responsibility for own medications	188	13	21	70	88.36	0.72	0.23
Ability to handle finances	147	50	23	72	75.00	0.47	0.002
**IADL according to the level of autonomy**							
8 IADL vs. 0	276	5	5	6	96.58	0.64	0.12
≥ 7 IADL vs. less	250	11	9	22	93.15	0.65	0.82
≥ 6 IADL vs. less	217	22	12	41	88.36	0.64	0.12
≥ 5 IADL vs. less	190	29	6	67	88.01	0.71	< 0.001
≥ 4 IADL vs. less	136	32	11	113	85.27	0.71	0.002
≥ 3 IADL vs. less	84	35	12	161	83.90	0.66	0.001
≥ 2 IADL vs. less	45	25	13	209	86.99	0.62	0.07
≥ 1 IADL vs. less	18	12	9	253	92.81	0.59	0.66
0 IADL vs. more	253	9	12	18	92.81	0.59	0.66

−/−: number of patients with no autonomy in face-to-face mode and no autonomy in telephone mode

−/+: number of patients with no autonomy in face-to-face mode and autonomy in telephone mode

+/−: number of patients with autonomy in face-to-face mode and no autonomy in telephone mode

+/+: number of patients with autonomy in face-to-face mode and autonomy in telephone mode

*Agreement was calculated as (*a* + *d*)/(*a* + *b* + *c* + *d*)

^†^*p* value of McNemar test

^‡^Linearly weighted Cohen’s kappa when more than 2 groups, i.e., IADL/8, sub-scores 1 and 2; unweighted Cohen’s kappa when 2 groups, i.e., IADL by item and IADL according to the level of autonomy

**Fig. 2:**
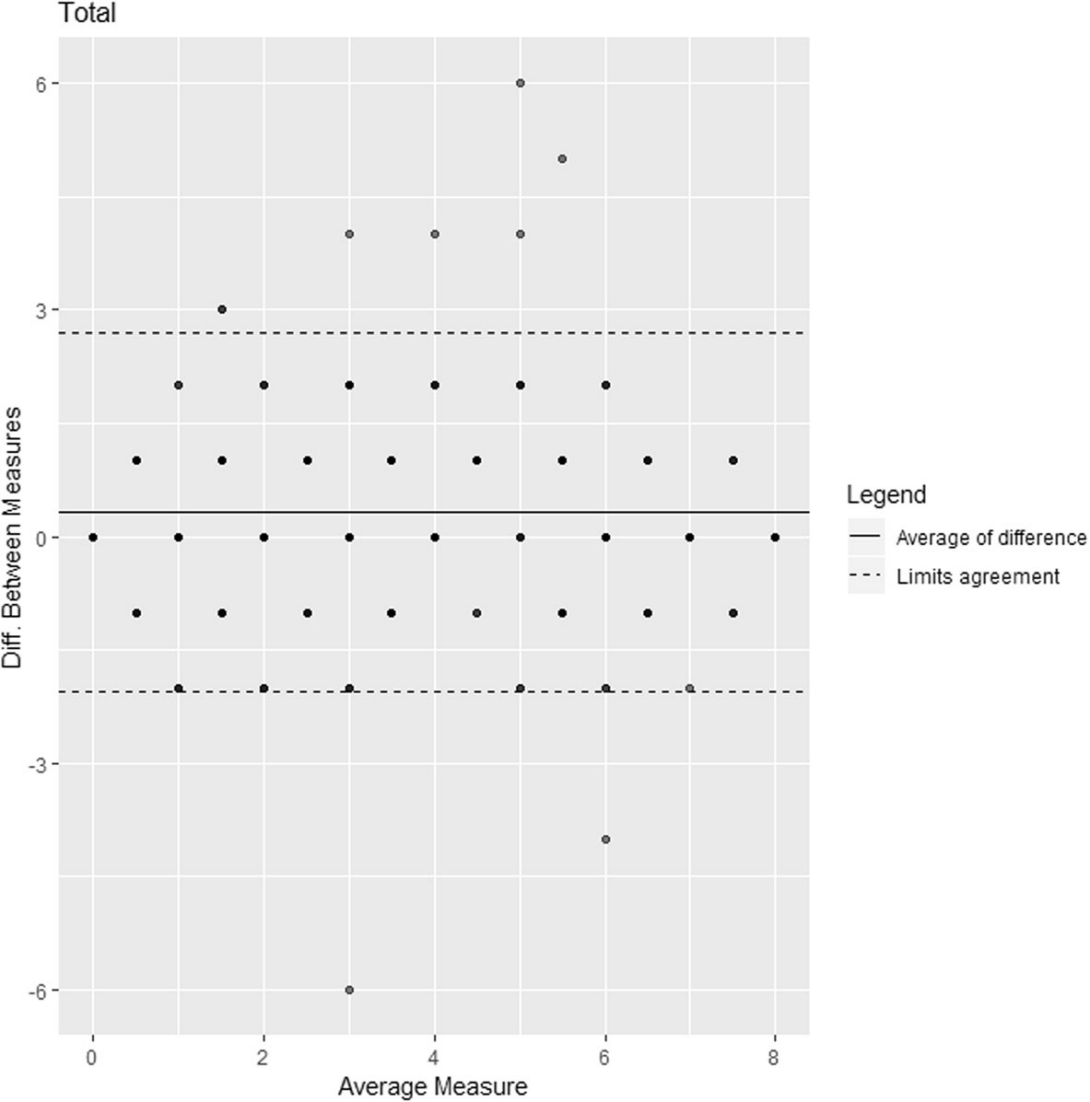
Bland-Altman plot to describe the agreement between the 2 modes of administration of the IADL questionnaire (by telephone and face-to-face)

Using the Bland and Altman method, the mean difference between the total IADL scores by telephone or face-to-face was 0.32 (Fig. 2). Out of the 292 patients, 96.9% lay within the 95% limits of agreement [− 2.06–2.70]. The mean difference between the total IADL scores by telephone or face-to-face was 0.27 in branch 1, 97.8% of the 134 patients lay within the 95% limits of agreement [− 2.10–2.64] (Supplementary files. Figure 1); and the mean difference between the total IADL scores was 0.37 in branch 2, 96.2% of the 158 patients were within the 95% limits of agreement [− 2.03–2.76] (Supplementary files. Figure 2).

### Factors associated with agreement between the 2 modes of administration

Investigation of the factors associated with the probability to lie within the limits of agreement found that age, sex, level of education, marital status, cognitive status, etiology, the MMSE, the type of relationship between the caregiver and the patient, a different respondent between the two assessments, the occurrence of death among family, or change of living place between the 2 assessments did not contribute significantly in the model (Table 3). Patients who were admitted to the emergency department, the occurrence of hospitalization, or a fall between the two assessments were less likely associated with the probability to lie within the limits of agreement. When included together in the same logistic regression model, only patients having experienced a fall remained less likely to be within the limits of agreement (OR = 0.07, 95% CI [0.02–0.27], *p* < 0.0001).

**Table 3:** Relationship between the characteristics of the patients and the odds to be included in the 95% limits of the Bland and Altman agreement

	OR	95% CI	***p*** value
**Study branch**			
1	Ref.		
2	0.58	0.14–2.37	0.45
**Age (year)**	1.06	0.75–1.16	0.17
**Sex**			
Female	Ref.		
Male	0.34	0.08–1.37	0.13
**Education**			
≤ 12 years	Ref.		
> 12 years	0.60	0.11–3.39	0.60
Unknown	0.50	0.11–2.28	0.37
**Marital status**			
Married/in couple	Ref.		
Single/widowed	3.50	0.40–30.50	0.26
Divorced/separated/other/unknown	1.10	0.26–4.72	0.90
**Cognitive status**			
Isolated memory complaint	Ref.		
Mild neurocognitive disorders	2.31	0.23–22.74	0.47
Major neurocognitive disorders	2.19	0.36–13.43	0.40
No neurocognitive disorders/unknown	0.40	0.08–2.08	0.27
**Etiology**			
Probable Alzheimer’s disease	Ref.		
Other neurological diseases	2.18	0.26–18.48	0.47
Awaiting diagnosis	0.73	0.14–3.76	0.71
**MMSE** (*n* = 250)	0.92	0–78-1.07	0.27
**Number of calls**	0.71	0.29–1.71	0.44
**Relationship between the caregiver and the patient at baseline**			
Spouse	Ref.		
Child	1.40	0.31–6.40	0.66
Other	0.31	0.05–1.98	0.22
**Change of respondent between the 2 assessments**			
No	Ref.		
Yes	0.20	0.02–1.85	0.16
**Events between the 2 assessments**			
Entry in emergency department (ref. no entry)	0.12	0.02–0.63	0.01
Occurrence of an hospitalization (ref. none hospitalization)	0.16	0.03–0.87	0.03
Occurrence of a fall (no fall)	0.07	0.02–0.27	< 0.001
Occurrence of a death in family (no occurrence)	–*	–*	0.99
Change of living place (ref. no change)	–*	–*	0.99

*OR was not calculated due to cells at 0

## Discussion

In this randomized crossover study conducted in a MC, we compared “face-to-face” to “telephone” administration of the French version of Lawton IADL questionnaire among caregivers and found that these provide similar assessment of the functional level of the patients when there were no events potentially influencing the score between the measurements, i.e., falls, admission to an emergency department, or hospitalization. When comparing the total IADL score with both modes of administration, the analysis by kappa coefficients, ICC, and with the Bland and Altman method found moderate to excellent agreement, with approximately 97% of the sample lying within the 95% limits of agreement. Another interesting result of this study is that the agreement was not influenced by the baseline characteristics of the patients, in particular cognitive impairment. We expected this result as the administration of the IADL questionnaire was performed with a proxy respondent, i.e., the caregivers and not the patient might explain this result [19].

### Strengths and weaknesses of the study

To our knowledge, this is the first study comparing telephone to face-to-face administration of the Lawton IADL. The randomized crossover design of the study allowed to demonstrate that the order of administration did not influence the magnitude of agreement. The collection of data was conducted both specifically for the present study and as part of the MEMORA study in order to reduce the cost of carrying out the study. The present study was completed by collection of events that could occur between the assessments. Data were collected prospectively for patients visiting the MC for the first time which should avoid recall bias.

Loss of participants occurred before each assessment and the study was conducted among 69.5% of the selected participants, mainly either because the visit was canceled or postponed in branch 1 or because it was not possible to reach the caregiver in branch 2. These missing evaluations were expected in this population, the patient having at a least memory complaint and the caregiver possibly experiencing burden making participation difficult [20]. As we anticipated this loss, the sample size was corrected a priori; nevertheless, it constitutes an attrition bias. It was obviously not possible to evaluate the agreement between patients without both IADL measurements but we compared patient characteristics according to completeness of IADL data at the second assessment and slight differences were found in terms of education, marital status, and cognitive status. Nevertheless, these differences were due to a higher proportion of unknown data among patients not included because face-to-face interview was not performed. In any case, since patient’s characteristics were not associated with a difference between the IADL measures, we believe that these missing data did not influence the results. If the drop-out was due to an event having an impact on the functional abilities of the patient, such as a fall, admission to emergency department, or a hospitalization, this could have led to lower agreement.

The proportion of participants unreachable by telephone was higher in branch 1, when the telephone interview was planned after the face-to-face interview, than in branch 2 in which telephone interview was planned first. As we could not reach the participants, we could not collect the reason of a non-response. However, we can speculate that this difference between the branches could be due to different delays between the first contact with the study nurses and the telephone interview, i.e., in the branch 1, the telephone interview was planned 1 month earlier to collect IADL, while it was planned with a shorter delay prior the face-to-face interview in branch 2. Indeed, in a context of medical appointment, it has been reported that no-show rates increase with increasing time between scheduling and the actual appointment [21].

### Comparison with the literature

The present study extends results of previous investigations that have compared telephone and face-to-face interviews for the evaluation of cognitive impairment in elderly people with various scales, and which generally found that telephone interview provides an adequate method to collect data [6, 22]. Nevertheless, we noted that prior studies often included small sample sizes, and they often used correlation to compare the different modes of administration instead of studying the degree of agreement which is a more appropriate methodology to achieve the objective. In the study reported by Monteiro et al. that included 30 elderly subjects, the authors found that compared to face-to-face interview, telephone interview provided a reliable measure of functional status evaluated through the functional assessment staging tool [23] with an ICC > 0.9 [7].

While previous studies generally assessed the comparison of different modes of administration of questionnaires among patients themselves, the administration of the IADL questionnaire to caregivers instead of the patients themselves in our study is justified for those who may experience cognitive impairment. Indeed, previous studies found that answers by patients may be influenced by their cognitive status and the presence of behavioral disorders [24, 25]. In addition, we found that the duration of telephone interviews to assess the IADL was faster on average (7 min) than administration during face-to-face interview (10 to 15 min) [26].

The effect of falls and, to a lesser extent, hospitalization and admission to an emergency department on the degree of agreement between the two modes of administration of the IADL questionnaire was not surprising as these events can be associated with a reduction in functional abilities or be a marker of health conditions leading to functional impairment [27].

## Conclusions

The results of the present study provide evidence that the administration of Lawton IADL questionnaire by telephone with a primary caregiver is acceptable for MC patients in comparison to face-to-face interview. The events that have occurred in a time close to the evaluation should be reported. The administration of the Lawton IADL questionnaire by telephone could therefore be implemented in clinical practice in order to improve the completeness of functional autonomy assessment.

## Supplementary information

**Additional file 1: Table S1.** Comparison of the characteristics of the patients included to those not included in the study. **Table S2.** Description of agreement between the 2 modes of administration of the IADL questionnaire by branch. **Figure S1.** Bland-Altman plot to describe the agreement between the 2 modes of administration of the IADL questionnaire (by telephone and face-to-face) in branch 1. **Figure S2.** Bland-Altman plot to describe the agreement between the 2 modes of administration of the IADL questionnaire (by telephone and face-to-face) in branch 2.

## Data Availability

The datasets generated and/or analyzed during the current study are not publicly available due to the French regulation on research but are available from the corresponding author on reasonable request.

## Abbreviations

eCRF: Electronic case report form
IADL: Instrumental Activities of Daily Living
ICC: Intraclass correlation coefficient
MC: Memory clinic
MMSE: Mini-Mental State Examination
NCD: Neurocognitive disorder
