# Supplementary material for: Comparison of Instrumental Activities of Daily Living assessment by face-to-face or telephone interviews: a randomized, crossover study
Source: Alzheimers Res Ther. 2020 Mar 13;12:24. doi: 10.1186/s13195-020-00590-w (PMC7068883; doi:10.1186/s13195-020-00590-w)

**S. Table 1 Comparison of the characteristics of the patients included to those not** included in the study

|  | **Total (n=365)** | **Patients included (n=292)** | **Patients not included (n=73)** | **p** |
| --- | --- | --- | --- | --- |
| **Age (years)** - Mean ± SD | 81.58 ± 7.18 | 81.51 ± 6.95 | 82.33 ± 8.08 | 0.72 |
| **Sex** - n (%) |  |  |  |  |
| Female | 224 (61.4%) | 172 (58.9%) | 52 (71.2%) | 0.053 |
| Male | 141 (38.6%) | 120 (41.1%) | 21 (28.8%) |  |
| **Education** - n (%) |  |  |  |  |
| ≤ 12 years | 202 (55.3%) | 173 (59.2%) | 29 (39.7%) | <0.001 |
| > 12 years | 61 (16.7%) | 53 (18.2%) | 8 (11.0%) |  |
| Unknown | 102 (27.9%) | 66 (22.6%) | 36 (49.3%) |  |
| **Marital status**  n (%) |  |  |  |  |
| Married/in couple | 146 (39.7%) | 125 (42.8%) | 20 (27.4%) | 0.001 |
| Single/widowed | 101 (27.7%) | 85 (29.1%) | 16 (21.9%) |  |
| Divorced/separated/other/unknown | 119 (32.6%) | 82 (28.1%) | 37 (50.7%) |  |
| **Cognitive status** – n (%) |  |  |  |  |
| Isolated memory complaint | 96 (26.3%) | 81 (27.7%) | 15 (20.5%) | <0.001 |
| Mild neurocognitive disorders | 71 (19.5%) | 61 (20.9%) | 10 (13.7%) |  |
| Major neurocognitive disorders | 135 (37.0%) | 116 (39.7%) | 19 (26.0%) |  |
| No neurocognitive disorders / Unknown | 63 (17.3%) | 34 (11.6%) | 29 (39.7%) |  |
| **Etiology** - n (%) (n=352) |  |  |  |  |
| Probable Alzheimer's disease | 76 (20.8%) | 65 (22.3%) | 11 (15.1%) | 0.37 |
| Others neurological diseases | 56 (15.3%) | 45 (15.4%) | 11 (15.1%) |  |
| Awaiting diagnosis | 233 (63.8%) | 182 (62.3%) | 51 (69.9%) |  |
| **MMSE** - Mean ± SD (n=293) | 19.56 ± 6.16 | 19.58 ± 6.14 | 19.44 ± 6.34 | 0.90 |
| **IADL at first assessment*** - Mean ± SD | 3.49 ± 2.23 | 3.45 ± 2.13 | 3.67 ± 2.61 | 0.50 |
| **Branch** - n (%) |  |  |  |  |
| 1 | 160 (43.8%) | 134 (45.9%) | 26 (35.6%) | 0.11 |
| 2 | 205 (56.2%) | 158 (54.1%) | 47 (64.4%) |  |
| * by phone or in-person according to study branch | | |  |  |

S.Table 2 Description of agreement between the 2 modes of administration of the IADL questionnaire by branch

|  | **Branch 1 (n=134)** | | | | | | | **Branch 2 (n=158)** | | | | | | |
| --- | --- | --- | --- | --- | --- | --- | --- | --- | --- | --- | --- | --- | --- | --- |
|  | **n**  **-/-**  **(a)** | **n**  **-/+**  **(b)** | **n +/- (c)** | **n +/+ (d)** | **Agreement^*^**  **%** | **K^‡^** | **p^†^** | **n**  **-/-**  **(a)** | **n**  **-/+**  **(b)** | **n +/- (c)** | **n +/+ (d)** | **Agreement^*^**  **%** | **K^‡^** | **p^†^** |
| IADL (/8) |  |  |  |  | 89.74% | 0.66 | <0.001 |  |  |  |  | 89.08% | 0.66 | <0.001 |
| Sub-score 1 (/4) - phone, transportation, medications, finance |  |  |  |  | 91.60% | 0.75 | <0.001 |  |  |  |  | 87.66% | 0.64 | <0.001 |
| Sub-score 2 (/4) - shopping, food, housekeeping, laundry |  |  |  |  | 85.63% | 0.59 | <0.001 |  |  |  |  | 86.08% | 0.63 | <0.001 |
| **IADL by item (autonomy Yes vs. No)** |  |  |  |  |  |  |  |  |  |  |  |  |  |  |
| Ability to use phone | 18 | 2 | 7 | 107 | 93.28 | 0.76 | 0.18 | 15 | 8 | 7 | 128 | 90.51 | 0.61 | 0.99 |
| Shopping | 103 | 12 | 2 | 17 | 89.55 | 0.65 | 0.01 | 109 | 15 | 13 | 21 | 82.28 | 0.49 | 0.85 |
| Food preparation | 98 | 14 | 3 | 19 | 87.31 | 0.62 | 0.01 | 111 | 17 | 5 | 25 | 86.08 | 0.61 | 0.02 |
| Housekeeping | 29 | 22 | 14 | 69 | 73.13 | 0.42 | 0.24 | 37 | 19 | 9 | 93 | 82.28 | 0.60 | 0.09 |
| Laundry | 62 | 13 | 11 | 48 | 82.09 | 0.64 | 0.84 | 75 | 17 | 5 | 61 | 86.08 | 0.72 | 0.02 |
| Mode of transportation | 88 | 7 | 8 | 31 | 88.81 | 0.73 | 0.99 | 82 | 16 | 3 | 57 | 87.97 | 0.76 | 0.004 |
| Responsability for own medications | 89 | 8 | 9 | 28 | 87.31 | 0.68 | 0.99 | 99 | 5 | 12 | 42 | 89.24 | 0.75 | 0.14 |
| Ability to handle finances | 68 | 20 | 8 | 38 | 79.10 | 0.56 | 0.04 | 79 | 30 | 15 | 34 | 71.52 | 0.39 | 0.04 |
| **IADL according to the level of autonomy** |  |  |  |  |  |  |  |  |  |  |  |  |  |  |
| 8 IADL vs. 0 | 128 | 2 | 1 | 3 | 97.76 | 0.67 | 0.27 | 148 | 3 | 4 | 3 | 95.57 | 0.61 | 0.38 |
| >=7 IADL vs. Less | 116 | 8 | 3 | 7 | 91.79 | 0.52 | 0.23 | 134 | 3 | 6 | 15 | 94.30 | 0.74 | 0.51 |
| >=6 IADL vs. Less | 104 | 9 | 4 | 17 | 90.30 | 0.67 | 0.27 | 113 | 13 | 8 | 24 | 86.71 | 0.61 | 0.38 |
| >=5 IADL vs. Less | 95 | 11 | 2 | 26 | 90.30 | 0.74 | 0.02 | 95 | 18 | 4 | 41 | 86.08 | 0.69 | 0.004 |
| >=4 IADL vs. Less | 65 | 16 | 6 | 47 | 83.58 | 0.67 | 0.052 | 71 | 16 | 5 | 66 | 86.71 | 0.74 | 0.03 |
| >=3 IADL vs. Less | 39 | 15 | 7 | 73 | 83.58 | 0.65 | 0.13 | 45 | 20 | 5 | 88 | 84.18 | 0.66 | 0.004 |
| >=2 IADL vs. Less | 24 | 8 | 8 | 94 | 88.06 | 0.67 | 0.99 | 21 | 17 | 5 | 115 | 86.08 | 0.57 | 0.02 |
| >=1 IADL vs. Less | 9 | 4 | 6 | 115 | 92.54 | 0.60 | 0.75 | 9 | 8 | 3 | 138 | 93.04 | 0.58 | 0.23 |
| 0 IADL vs. More | 115 | 6 | 4 | 9 | 92.54 | 0.60 | 0.75 | 138 | 3 | 8 | 9 | 93.04 | 0.58 | 0.23 |

-/- : number of patients with no autonomy in in-person mode and no autonomy in phone mode;

-/+ : number of patients with no autonomy in in-person mode and autonomy in phone mode;

+/- : number of patients with autonomy in in-person mode and no autonomy in phone mode;

+/+ : number of patients with autonomy in in-person mode and autonomy in phone mode.
^*^ Agreement was calculated as : (a+d)/(a+b+c+d)

**^†^** p value of Mac Nemar test

**^‡^** Linearly weighted Kappa coefficients when more than 2 groups i.e. IADL/8, sub-scores 1 and 2.

**s. Figure 1 Bland-Altman plot to describe the agreement between the 2 modes of administration of the IADL questionnaire (by telephone and face-to-face) in branch 1**

**
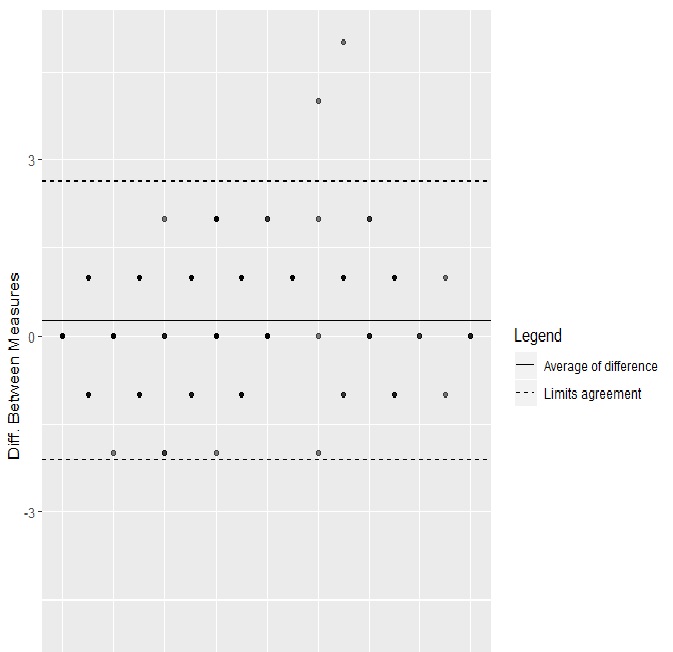
**

**s. Figure 2 Bland-Altman plot to describe the agreement between the 2 modes of administration of the IADL questionnaire (by telephone and face-to-face) in branch 1**


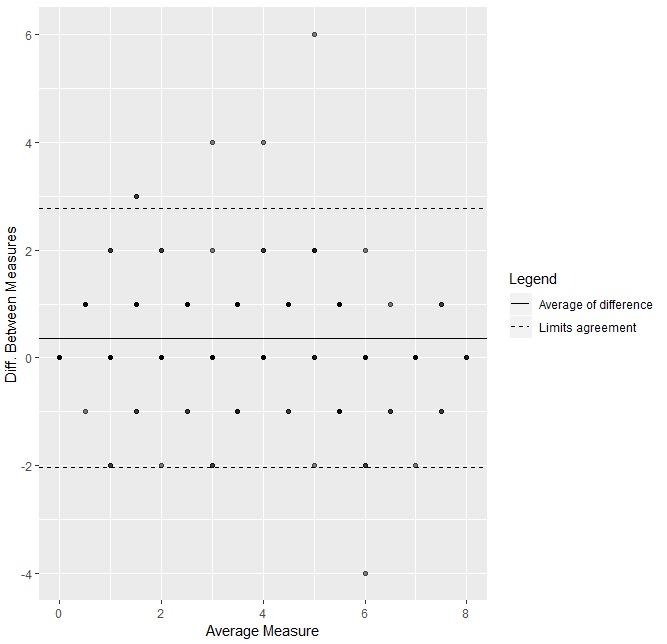

Supplement: Supplementary file 1 — Additional file 1: Table S1. Comparison of the characteristics of the patients included to those not included in the study. Table S2. Description of agreement between the 2 modes of administration of the IADL questionnaire by branch. Figure S1. Bland-Altman plot to describe the agreement between the 2 modes of administration of the IADL questionnaire (by telephone and face-to-face) in branch 1. Figure S2. Bland-Altman plot to describe the agreement between the 2 modes of administration of the IADL questionnaire (by telephone and face-to-face) in branch 2. [file 13195_2020_590_MOESM1_ESM.doc]
